# Impact of the Immunogen Nature on the Immune Response against the Major HBV Antigens in an HBsAg and HLA-humanized Transgenic Mouse Model

**DOI:** 10.5005/jp-journals-10018-1094

**Published:** 2014-01-22

**Authors:** M Mancini-Bourgine, G Guillen, ML Michel, JC Aguilar

**Affiliations:** 1 Laboratoire de Pathogenese des virus de l’hepatite B, Institut Pasteur, Paris, France; Inserm U845, Unite de Pathogenese des hepatites virales B et Immunotherapie, Paris, France; 2 Vaccine Division, Clinical Trials Department, Center for Genetic Engineering and Biotechnology, Havana City, Cuba; 3 Laboratoire de Pathogenese des virus de l’hepatite B, Institut Pasteur, Paris, France; Inserm U845, Unite de Pathogenese des hepatites virales B et Immunotherapie, Paris, France; 4 Vaccine Division, Clinical Trials Department, Center for Genetic Engineering and Biotechnology, Havana City, Cuba

**Keywords:** Immunogen, Transgenic mice, HBsAg, HBV, HBcAg, Humoral immunity, Cellular immunity.

## Abstract

Hepatitis B chronic carriage remains as a major public health problem. Protein and DNA vaccines are now widely used in therapeutic vaccine candidates. Although, the hepatitis B surface antigen (HBsAg) based vaccines have been largely studied, candidates comprising both HBsAg and core (HBcAg) either protein- or DNA-based approaches deserve further immunological characterization.

In the present study, a repeated dose administration schedule for protein or DNA immunogens was conducted in order to characterize the resulting immune response in a humanized and HBsAg-tolerized setting. A novel transgenic (Tg) mice that express the HBsAg, human MHC class I (HLA-A*0201) and MHC class II (HLA-DRB1*01) molecules and devoid of endogenous murine class I and II molecules was used as a model of HBV chronic carrier. Mice were immunized by subcutaneous (protein) or intramuscular (DNA) routes and the humoral and cellular responses were evaluated.

Protein or DNA immunization induced humoral immune responses against both HBsAg and HBcAg. The systematic analysis of epitopes that activate CD4+ and CD8+ T lymphocytes confirmed the accuracy of the model. Cellular immune responses were detected differing in their nature. CD8 T-cell responses were induced mostly after DNA immunization while CD4 T-cell responses were predominant in protein based immunizations. In addition, the intensity of HLA-A2-restricted CD8+ T cell responses was reduced in Tg mice expressing HBsAg when compared to control Tg mice.

In conclusion, our results indicate that cellular immune responses necessary for the development of protective immunity can be achieved by DNA or protein immunization. However, important differences in their nature arise when immunogens are administered several times.

**How to cite this article:** Mancini-Bourgine M, Guillen G, Michel ML, Aguilar JC. Impact of the Immunogen Nature on the Immune Response against the Major HBV Antigens in an HBsAg and HLA-humanized Transgenic Mouse Model. Euroasian J Hepato-Gastroenterol 2014;4(1):36-44.

## INTRODUCTION

Hepatitis B virus (HBV) chronic infection remains a major public health problem. More than one-third of the world population has been infected by the HBV, resulting in more than one million deaths every year as a result of a progressive hepatic disease and their complications.^1^

Therapeutic vaccination has been evaluated in the treatment of chronic HBV infection. The fundamental role of the immune response in controlling the HBV constitutes the rationality of this approach. The target is to subvert the HBV immune-tolerance by therapeutic vaccination, but their development has proven to be difficult.^2^ More potent vaccine candidates including new antigens or adjuvants as well as the study of new administration routes and the combination of immunotherapy and antivirals are required. A large clinical trial evaluating the hepatitis B surface antigen (HBsAg) in formulation with a strong adjuvant suggested the use of hepatitis B care antigen (HBcAg) as the election antigen in addition to HBsAg to improve the formulation.^3^

The recombinant HBcAg VLP has attractive physico-chemical characteristics leading to important immunological properties, such as: (a) capacity to simultaneously behave both as a T-dependent and T-independent antigen,^5^ (b) superior immunogenicity compared to their soluble antigenic form (HBeAg), estimated as 1000 times,^6^ (c) capacity to induce stronger Th1 response as compared to HBeAg that mainly promote a Th2-like response,^7^ (d) the capacity to help not only HBcAg-specific B cells but also anti-HBsAg responses,^8^ and (e) the capacity to be used as a carrier protein able to improve conjugated or inserted antigens.^9^

Particulated HBcAg, produced in *E. coli* as a nucleo-protein, enhances the immune response to coadministered HBsAg or heterologous antigens, modulating the resulting immune response in a Th1 sense, the type of immune response required for the development of new therapeutic vaccine candidates.^10-12^ This vaccine is currently under clinical investigation of phase III clinical trial after evidencing to be safe and immunogenic in healthy volunteers and chronically infected patients.^13,14^

DNA-based vaccines have been used to boost or to broaden the weak virus-specific T-cell response during the chronic hepatitis B infection. A DNA vaccine expressing HBV small (S) and middle (preS2 +S) envelope proteins is currently under clinical investigation. A phase I clinical trial in chronic HBV carriers showed evidences for the safety and immunogenicity of HBV-DNA vaccination and demonstrated that DNA vaccination can restore or activate T-cell responses in chronic HBV carriers.^15^ DNA vaccination can specifically but transiently activate T-cell responses in some chronic HBV carriers who do not respond to current antiviral therapies.^16^

The H-2 class II deficient mouse that express the human MHC class II molecule, HLA-DR1, has been used to identify novel hepatitis B virus T-cell epitopes.^17^ An HBsAg/ HLA-A2 humanized murine model was developed to study T cell tolerance to the HBsAg that is present in sera of HBV chronic carriers. The double-transgenic mice express a chimeric HLA-A2 MHC class I molecule and a high amount of the HBsAg in the liver that is secreted and present in sera during the animal’s lifetime.^18^

In the present study, HBs-A2-DR1 mice were immunized seven times with a DNA- or protein-based vaccine in order to explore the resulting cellular or humoral immunity against both, HBsAg and HBcAg, in this special mice model following a more intense immunization schedule.

## MATERIALS AND METHODS

### Protein Antigens

HBsAg was produced to more than 95% purity at the Center for Genetic Engineering and Biotechnology production facilities (CIGB, Havana, Cuba) as a component of the commercial HBV prophylactic vaccine, Heberbiovac-HB. HBsAg for this vaccine is expressed and purified from the yeast Pichia pastoris. The entire core antigen (HBcAg) expressed in *E. coli* strain W3110 was obtained with a purity of >95% under GMP conditions at the CIGB. The formulation containing both antigens at (1:1) proportion was obtained by simple mixture in phosphate buffer (8 mM, EDTA 6 mM, NaCl 140 mM pH 6.8).

### DNA Vectors

pCMV-S2.S^19^ encodes the small (S) and middle (preS2 + S) proteins of the HBV envelope (ayw subtype) under the control of the cytomegalovirus (CMV) immediate early gene promoter. The pMAS core plasmid (a kind gift of HL Davis) was used as the DNA encoding for the HBV capsid. The plasmid DNA used for immunization was purified using anion exchange chromatography columns (Plasmid Factory, Bielefeld, Germany), dissolved in endotoxin-free PBS (Sigma, St Louis, MO) at 1 mg/ml and stored at or below -20°C until use.

### Synthetic Peptides

Four distinct sets of synthetic peptides were used in this study: peptides containing known HLA-A2- and HLA-DR1-binding motifs within the S domains of the HBV envelope proteins; a pool of ten 15-mer peptides spanning the total preS2 region of HBV envelope subtype ayw; 9 to 15-mer peptides spanning the entire region of HBV envelope subtype ayw and adw were divided into four sets of eleven peptides (S164-218, S213-286, S285-357 and S338-389), and thirty-six HBV core 15-mer overlapping peptides comprising the whole HBV core antigen for genotype A, which shares all anchor areas within the immunodominant epitopes with the other genotypes were divided into four sets of nine peptides (C 1-55, C46-95, C86-140 and C131-185, also named as C1A, C1B, C2A and C2B, respectively).

Synthetic peptides were purchased from polypeptide Group (Strasbourg, France). Peptide purity was > 80%. Peptides were dissolved in RPMI or DMSO at a concentration of 10 mg/ml and diluted before use at 1 to 2 ng/ml with culture medium.

### Mice

**Fig. 1:**
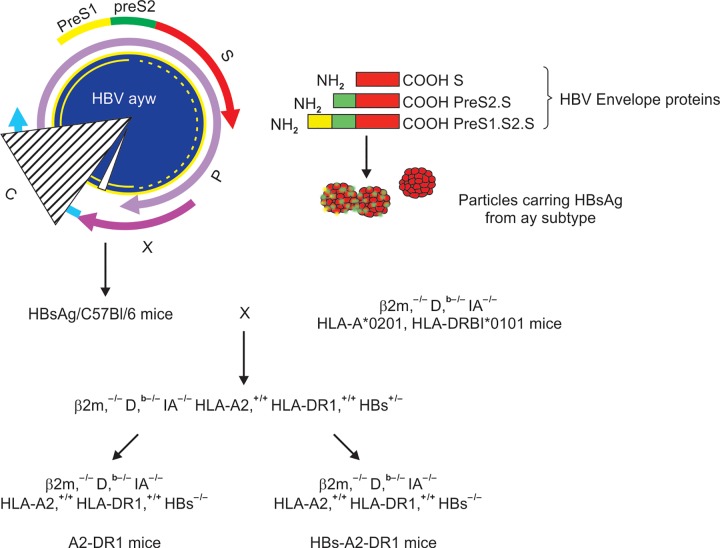
New animal model of chronic HBV carriers. The previous transgenic mouse model (Mancini M. et al, Proc. Natl. Acad. Sci, USA 1996 = Ref 20 was improved by creating transgenic mice that express the Hepatitis B surface antigen (HBsAg), human MHC class I (HLA-A*0201) and MHC class II (HLA-DRB1*0101) molecules that are devoid of endogenous murine class I (P2m and D^b^) and class II (IAb) molecules. Due to the presence of the HBsAg transgene, this new mouse lineage expresses high levels of HBsAg ay in the liver that is secreted into the serum and present at the periphery throughout life. P, X, C: polymerase, X, core open reading frames, respectively

A new animal model of chronic HBV was obtained. A former transgenic mouse model ^18,20,21^ was improved by creating transgenic mice that express the HBsAg, human MHC class I (HLA-A*0201) and MHC class II (HLA-DRB1*0101) molecules. This mouse model is devoid of endogenous murine class I (P2m and D^b^) and class II (IAb) molecules. Due to the presence of the HBsAg transgene, this new mouse lineage expresses high levels of HBsAg in the liver that is secreted into the serum and present at the periphery throughout life (Fig. 1). Briefly, the novel transgenic mouse lineage was obtained by intercrossing HLA-A*0201^+/+^/HLA-DR1^+/+^ double-transgenic H-2 class I (β2m^0/0^)-/class II (IAβ^0/0^)-KO animals^21^ and HBsAg^+/0^/HLA-A*0201^+/+^ (β2m^0/0^/IAβ^0/0^).^18^ Progeny were first screened by PCR for the presence of the HLA-A*0201 and HLA-DR1 transgenes until HBsAg^+/0^/HLA-A*0201^+/+^/HLA-DR1^+/+^ transgenic H-2 class I (β2m^0/0^)-/class II (IAβ^0/0^)-KO animals were obtained. The homozygous status of HLA-A*0201 and HLA-DR1 transgenes was confirmed by the presence of both transgenes in the progeny obtained by crossing the novel transgenic mice with C57BL/6 mice. The HBsAg/ HLA-A2/HLA-DR1 transgenic mice express chimeric HLA-A2 MHC class I, HLA-DR1 MHC class II molecules and HBV envelope proteins carring the HBsAg of genotype D subtype ayw in the liver, which are secreted and present in sera during the animal’s lifetime. Due to the heterozygous status of the HBV transgene only 50% of offsprings express the HBV transgene and will thereafter referred as HBs-A2-DR1 and A2-DR1 mice depending on whether they express or not HBsAg.

All mice were 8 to 12 weeks of age at the first administration and were maintained at Pasteur Institute animal facility, under specific-pathogen-free conditions. All protocols were reviewed by the Institut Pasteur competent authority for compliance with the French and European regulations on Animal Welfare and with Public Health Service recommendations.

### Immunization Schedule

**Fig. 2:**
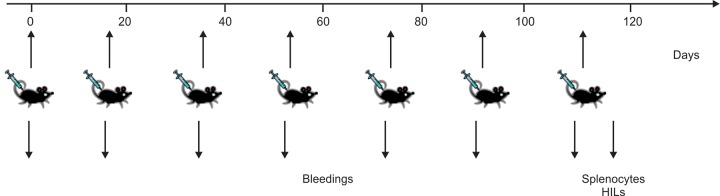
The immunization schedule was designed to evaluate seven inoculations of two types of immunogens. One of the immunogens, NASVAC, is a vaccine formulation consisting of a mixture of yeast-derived recombinant HBsAg ad and *Escherichia coli* purified recombinant full length HBcAg. A total of 10 ug of NASVAC (5 mg HBsAg ad + 5 ug HBcAg) in Alum, was administered by SC route, a second group of mice was pretreated by cardiotoxin 5 days before inoculation with 50 ug of pCMV-S2.S ad and 50 ug of pMasCore DNAs by the IM route. Mice were bled before each injections and one week after the last injection at the time of sacrifice

The immunization schedule was designed to evaluate seven inoculations of two types of therapeutic vaccine candidates: (a) NASVAC, a formulation consisting of a 1:1 mixture of yeast-derived recombinant HBsAg (ad) and the *Escherichia coli* purified recombinant full length HBcAg and (b) the mixture of two DNAs coding for the same antigens. Every 17 days, a total of 10 ng of NASVAC (5 ng HBsAg ad + 5 ng HBcAg) was administered in Alum by the SC route while a second group of mice was inoculated with 50 ***\i****g* of pCMV-S2.S ad and 50 ng of pMasCore DNAs by the IM route. Placebo controls were inoculated either with alum or PBS only and used as negative groups for ELISA and cellular studies. A total of five mice were used in each experimental procedure with both types of mice, HBs-A2-DR1 and A2-DR1 transgenic controls (Fig. 2).

### Biological Fluids and Serology

Blood samples were collected before and 2 weeks after each immunizations through the retro-orbital plexus and centrifuged at 1500 xg for 10 minutes (Eppendorf centri-fugue, Hamburg, Germany). The sera were collected and stored at -20°C until evaluation as previously described.^22^

Serum HBsAg Level

The sera from immunized or nonimmunized HBs-A2-DR1 mice were tested for detection of HBsAg by a commercial ELISA kit (Monolisa AgHBs Plus; Bio-Rad, Marnes la Coquette, France).

Total anti-HBsAg/anti-HBcAg IgG

At various times before and after vaccination, antibodies were quantified in sera by ELISA as previously reported.^22^ HBV core antigen (HBcAg) and particles of HBsAg ‘a’ group and ‘d’ subtype (identical to the injected products) or particles of PreS2-HBs ay ‘a’ group and ‘y’ subtype (expressed by the Tg mice), were used to detect anticapsid and antienvelope antibodies, respectively. Antibody titers were determined by serial end-point dilutions. The titer was defined as the highest serum dilution that resulted in an absorbance value two times greater than that of nonimmune serum. The limit of detection for antibody titers is indicated by a dashed area in each figure.

### Cellular Immunity

ELISPOT Assay

Splenocytes (0.5 × 10^6^ cells/well) were isolated from immunized mice and incubated with peptide pools (2 ug/ml of each peptide) in complete a-MEM medium supplemented with 10% FCS onto sterile nitrocellulose MSIP 96-well plates (Millipore, Bedford, MA) coated with capture antibodies against mouse gamma interferon (IFN-γ). The IFN-γ coated plates were incubated at 37°C for 18 hours before scoring the number of spots with a Bioreader 4000 counter (Biosys). Splenocytes from non-immunized mice and cells in culture medium alone were used as negative controls to determine background levels. Each cell population was titrated in triplicate. The response was considered positive if the median number of spots in triplicate wells was at least twice that observed in control wells containing medium, and at least 10 IFN-γ secreting cells per million splenocytes were detected.

### Intracellular Staining Protocol

For intracellular cytokine detection, freshly isolated sple-nocytes (1 × 10^6^ cells) were incubated for 1 hours either with medium alone or with peptides pools.^23^ Thereafter, brefeldin A (Sigma-Aldrich) was added to a final concentration of 2 ug/ml and the cultures were incubated overnight at 37°C. Cells were harvested, washed, and surface stained with anti-CD3, anti-CD4 and anti-CD8 conjugated mAb. Surface-stained cells were fixed with 2% paraformaldehyde in PBS. Fixed cells were resuspended in permeabilization buffer (PBS, 1% BSA, 0.05% saponin and 0.01% sodium azide) and incubated with PE-conjugated anti-IFN-γ mAb for 30 minutes at 4°C and washed twice in permeabili-zation buffer. Stained cells were resuspended in PBS/1% w/v BSA supplemented with 0.01% w/v sodium azide. All antibodies were purchased from BD Biosciences. Cells were acquired on FACSCanto (BD Biosciences) and frequencies were determined by flow cytometry analyses using Flowjo software (Tree Star).

### Statistical Procedures

Data were expressed as means ± SEM. Nonparametric unpaired comparisons were performed using the Mann-Whitney U test. For multiple comparisons of HBsAg concentrations before and after immunization, the Friedman test followed by Dunn’s post test was used. The significance level a was set 5%. Statistical analysis was carried out using Graphpad Prism 5 software. Values of p < 0.05 were considered significant.

## RESULTS

The present work is aimed at evaluating the immunogenicity of two formulations able to induce immunity against HBsAg and HBcAg in an HLA-humanized HBsAg transgenic mouse model. The immunization was designed to explore the effect of repeated dose administration of a DNA- or protein-based immunogens.

## SEROLOGY

### Anti HBcAg Ab Response

**Figs 3A to C:**
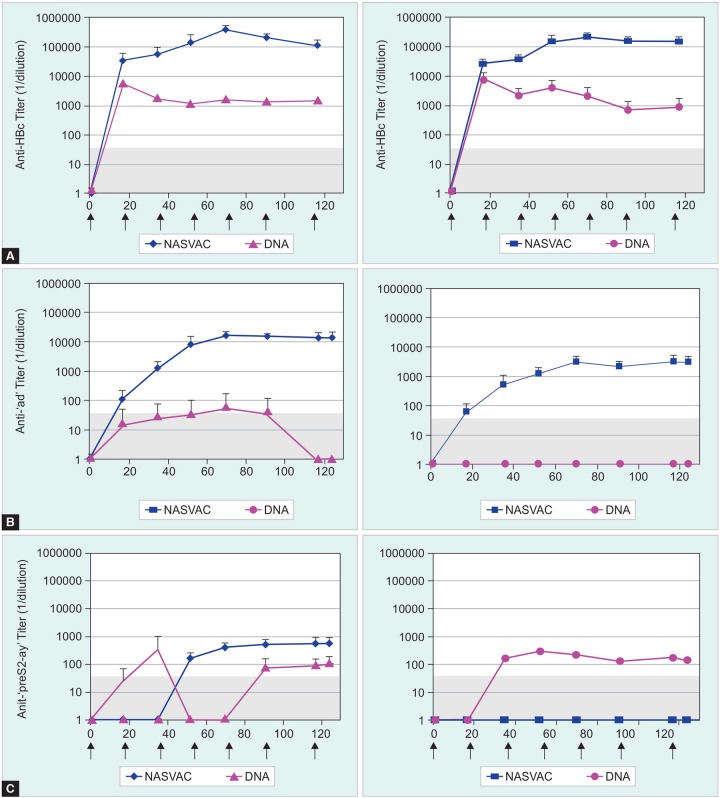
At various times before and after vaccination, antibodies were quantified in sera by ELISA. HBV core antigen (A) and (B) particles of HBsAg ‘a’ group and ‘d’ subtype (identical to the injected products) or (C) particles of PreS2-ay (‘a’ group and ‘y’ subtype expressed by the Tg mice), were used to detect anti-capsid and anti-envelope antibodies, respectively. Antibody titers were determined by serial end-point dilutions. The titer was defined as the highest serum dilution that resulted in an absorbance value two times greater than that of nonimmune serum. The limit of detection for antibody titers is indicated by a dashed area

Antibody responses against HBV envelope and capsid proteins are induced by both immunization regimens irrespective of the immunogen’s nature (Figs 3A to C). A similar anti-HBc antibody response in HBsAg positive compared to HBsAg negative mice was induced *(see* Fig. 3A). The antibody kinetics of the two lineages evidenced maximal anti-HBc titers 2 weeks after the first injection for DNA or 2 weeks after the fourth injection for NASVAC. At the peak of antibody production, anti-HBc titers were one hundred fold higher in NASVAC-immunized mice compared to DNA-immunized mice *(see* Fig. 3A). Although anti-HBcAg Ab response was similar after the first injection for both HBsAg positive or negative mice, anti-HBc titers continued to increase in NASVAC-immunized mice whereas there was a trend in the sense of reduction for the DNA candidate during the rest of the immunization schedule. This led, after 7 immunizations, to a higher intensity for protein immunizations.

### Anti HBsAg Ab Response

The HBsAg contained in the NASVAC formulation is less immunogenic than the core antigen since the anti-HBs antibody titers are tenfold lower than the anti-HBc antibody titers in both lineages *(see* Fig. 3B). According to differences observed in ELISA reactivity, anti-HBs antibodies are 90% subtype ‘d’ and 10% group-specific following NASVAC immunization of A2-DR1 mice (compare upper and lower panels). By contrast, only subtype-specific ‘d’ antibodies are detectable in HBs-A2-DR1 mice.

A strong difference in anti-HBs antibody titers was found between NASVAC and DNA-immunized mice.DNA immunization only generated anti-PreS2ay (see Fig. 3C) specific antibodies in A2-DR1 mice. These specific antibodies are also detectable in only one HBs-A2-DR1 mouse (mouse 6-16), suggesting that B-cell tolerance to envelope proteins can be subverted in this lineage *(see* Fig. 3B, lower right panel).

### HBsAg Serology

Despite anti-HBs antibody production, there were no significant differences in the levels of HBsAg in sera of HBsAg Tg mice between immunization groups before or after immunization. All HBs-A2-DR1 groups of mice had similar HBsAg levels at the end of the immunization schedule (data not shown).

### Cellular Immune Response

**Figs 4A to D:**
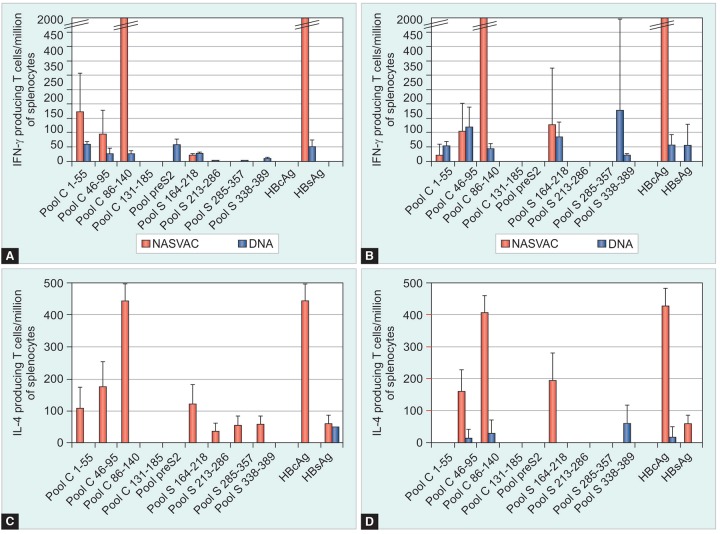
ELISPOT assay to determine the frequency of cells secreting IFNγ- (upper panels) or IL4 (lower panels). Splenocytes from A2-DR1 (left panels) and HBs-A2-DR1 (right panels) mice were stimulated with several pools of peptides panning the HBsAg and HBcAg protein sequences or HBsAg and HBcAg proteins. ELISPOT assays were conducted to evaluate the intensity of the cellular immune response after the immunization with protein (NASVAC, empty columns) or DNAs (pCMV-S2.S and pMasCore, black columns)

Cellular immunity was detected in A2-DR1 as well as in HBs-A2-DR1 mice (Figs 4A to D). In general, the intensity of the IFNγ- and IL4-producing T cell response after stimulation of the spleen cells with HBsAg or HBcAg proteins and peptides evidenced a superior response in the case of protein immunization compared with DNA immunization. Following NASVAC immunization, the IFNγ-producing T cells were predominantly directed against the core protein and more precisely against the pool of peptides C86-140 in both lineages *(see* Figs 4A to D upper panels). No response against the C-terminal part of the core protein was detected. By contrast, a weaker HBsAg-specific T-cell response was detected in A2-DR1 mice compared with HBs-A2-DR1 mice after protein immunizations. In HBs-A2-DR1 mice, two pools of peptides — pool S164-218 and S338-389—elicited a detectable IFNγ response irrespectively of the immunogen used. T-cell responses induced by DNA immunizations were similar in HBsAg-positive and negative mice and hardly detectable especially for IL-4 production.

The IFNγ positive results obtained in ELISPOT assays were refined by performing intracellular staining (Table 1). By using individual peptides, it was shown that NASVAC immunization induced a predominant CD4+ T cell response against HBcAg compared to the same antigens given as DNA immunogen. The only detectable response following DNA immunizations was a CD8+ T cell response against the peptide C16-30 which includes a well-defined HLA-A2 epitope. Regarding HBsAg, a CD4+ T cell response against peptide S200-214 of the HBsAg subtype ad was detected in the case of protein immunization. By contrast, the same peptide S200-214ad was recognized by CD8+ T cells after DNA immunization in both lineage of mice. IFNγ-positive intracellular staining of splenocytes restimulated with peptide S204-212 confirmed the presence of a CD8 epitope within the S200-214 sequence. Moreover, a broad CD8+ T cell response against HBsAg was induced in DNA-immu-nized A2-DR1 mice, including in addition to the peptide S204-212—the peptides S164-178 and S170-181. Although NASVAC was characterized by inducing strong CD4+ T cell responses, a CD8+ response was also detected in the HBs-A2-DR1 mice against the A2-restricted S348-357 epitope that is present in the S338-389 peptide pool (see Table 1). Response to this epitope was not induced after DNA immunization.

Present results confirm that DNA immunization induces mainly IFNγ-secreting CD8+ T cells while NASVAC immunization induces mainly IFNγ-secreting CD4+ T cells.

## DISCUSSION

Current therapies for CHB have resulted in a low therapeutic efficacy and considerable side effects,^24^ however, these drugs are the most effective therapies up to date. Therapeutic immunization has been explored in the last 20 years based on the role of antiviral immunity in HBV control. Clinical trials using HBsAg-based formulations have been inconclusive or evidenced the limitations of using HBsAg as the only antigen in the vaccine formulation.^3,25,26^ In summary, therapeutic immunization has resulted in a bigger challenge than expected. In line with this, the use of additional antigens (HBcAg), new adjuvant, and vaccination routes and schedules have been proposed.^3^

**Table 1:** **Table 1:** Identification of the specificity and phenotype of IFNγ- secreting T cells by intracellular staining. Splenocytes were collected 1 week after the last immunization. Individual peptides (2 |jg/ml final) derived from pools were used to stimulate splenocytes *ex vivo* for 1 hour prior to an overnight incubation with brefeldin A (2 jg/ml final). Cells were then stained with anti-CD3, -CD4 and -CD8 labeled antibodies. After fixation and permeabilization, intracellular IFNγ- detection was performed. ND: Not determined due to the lack of cells

						*NASVAC*								*DNAs*			
*Pools*		*A2-DR1*				*HBs-A2-DR1*				*A2-DR1*				*HBs-A2-DR1*			
		*Peptide*		*ICS*		*Peptide*		*ICS*		*Peptide*		*ICS*		*Peptide*		*ICS*	
C1-55		21-35		CD4		21-35		ND		ND		CD4 CD8		16-30		CD8	
C46-95		56-70		CD4		56-70		ND		ND		CD4 CD8		ND		ND	
		61-75		CD4		61-75											
C86-140		111-125		CD4		111-125		CD4		ND		CD4 CD8					
		115-130		CD4		115-130		CD4									
PreS2										164-128		CD4					
S164-218		200-214 ad		CD4		200-214 ad		CD4		164-178		CD8		164-178		ND	
										170-181		CD8		200-214 ad		CD8	
										200-214 ad		CD8		204-212 ad		CD8	
										204-212 ad		CD8					
S338-389						348-357		CD8									

The present evaluation of DNA and protein immunogens, in a repeated immunization schedule, evidenced differences in the resulting immune response that should be taken into consideration to optimize future therapeutic vaccination strategies.

The same level of HBsAg in the sera of protein or DNA immunized mice evidence that there is still space for further optimization as it has been previously shown that the HBsAg tg mice display a strong tolerogenic effect on the CD4(+) T cell compartment. This effect is associated with a defect in CD8(+) T cell effector functions in vivo and the inability of reducing the HBsAg levels.^18^

Mice receiving protein or DNA immunization induced humoral immune responses against both HBsAg and HBcAg. The increased level of anti-HBcAg Ab response after protein administration is in line with the previously described strong immunogenicity of HBcAg.^5-8^ The reduction in the Ab response after several DNA immunizations suggest that protein immunization may be more suitable for repeated immunization compared to DNA-based immunization.

Recent studies have used multiple administrations instead of traditional three to four dose schedules.^3,4^ Previous experience in HBsAg transgenic Balb/C mice and protein immunization suggest that the dynamic of tolerance subversion using protein immunogens require repeated administrations (5 or more) in order to subvert T or B-cell immune responses._27,28_

One of the greatest advantages of DNA vaccines is that they are able to induce cytotoxic T lymphocytes (CTL) without the inherent risk associated with living vaccines and with a high efficiency.^29^ In contrast to proteins expressed in the cytosol following DNA injection, the recombinant proteins used for immunization are endocytosed and presented on major histocompatibility complex class II molecules, facilitating CD4+ and humoral responses, explaining the CD4 oriented pattern of immune response of NASVAC and the CD8 biased profile of DNA immunization. However, NASVAC was able to induce a CD8+ immune response against peptide S (348-357) and previously in Balb/C mice against S (28-39). This CD8+ T cell response can be explained as a result of crosspriming process leading to class I presentation and immune response to the specific CD8+ T cell peptide.^30,31^

DNA prime and protein boost regimen or vice versa, may potentiate vaccine efficacy over that seen when only DNA or protein is administered. DNA vaccines consistently drive induction of strong CD8+ T-cell immune responses, while subunit protein vaccines elicit strong humoral responses and CD4+ T cell response. This combination may further increase both types of immune responses. The positive impact of protein boosting on DNA vaccine-specific immune responses has been demonstrated in literature.^32-37^ In addition, there are also evidences of the benefit of combining protein and DNA immunogens in the same formulation.^38^

The present results do not discard the potentialities of both vaccine candidates *per se.* Resolution of chronic hepatitis B and anti-HBsAg seroconversion in humans after adoptive transfer of immunity to hepatitis B core antigen^39^ support the results obtained by Al-Mahtab et al using NAS-VAC. The activating effect found after *ex vivo* NASVAC stimulation of dendritic cells and the antiviral effect after HBV Tg mice immunization with NASVAC-pulsed DC further support the clinical evaluation of NASVAC in the different scenarios.^40^

## CONCLUSION

Our results indicate that cellular immune responses necessary for the development of protective immunity can be achieved by DNA or protein immunization comprising the main HBV antigens in the ‘humanized’ Tg mice against both antigens. Anti-HBsAg T-cell responses were obtained in the HBsAg-transgenic mice using both types of antigens, evidencing the subversion of the antigen specific tolerance. Qualitative and quantitative differences in the resulting immune response arise when both types of immunogens are administered several times. Improved immunization strategies against HBV and other pathogens in which tolerance subversion is required are suggested combining both types of immunogens in rationally designed immunization schedules.
