# ERRTAUM

**Published:** 2014-07-28

In the last issue of Euroasian Journal of Hepato-Gastroenterology (EJOHG), that is , the article ‘Impact of the Immunogen Nature on the Immune Response against the Major HBV Antigens in an HBsAg and HLA-humanized Transgenic Mouse Model’ by M Mancini-Bourgine, G Guillen, ML Michel, JC Aguilar, was inadvertently omitted from the printed issue of the journal. Although being missed from the printed version, the same was included in the online version of the issue and can be read at http://www.jaypeejournals.com/eJournals/ShowText.aspx?ID=6229&Type=FREE&TYP=TOP&IN=~/eJournals/images/JPLOGO.gif&IID=471&isPDF=YES

